# RNA Sequencing Reveals Dynamic Carbohydrate Metabolism and Phytohormone Signaling Accompanying Post-mowing Regeneration of Forage Winter Wheat (*Triticum aestivum* L.)

**DOI:** 10.3389/fpls.2021.664933

**Published:** 2021-07-29

**Authors:** Guibin Cui, Mei Zhao, Hongbin Tan, Zhulin Wang, Min Meng, Fengli Sun, Chao Zhang, Yajun Xi

**Affiliations:** ^1^State Key Laboratory of Crop Stress Biology for Arid Areas, College of Agronomy, Northwest A&F University, Yangling, China; ^2^Key Laboratory of Wheat Biology and Genetic Breeding, Ministry of Agriculture and Rural Affairs, Yangling, China; ^3^Shaanxi Province Seed Industry Group Co., Ltd., Xi’an, China

**Keywords:** regeneration, forage wheat, mechanical injury, pasture, mowing, carbohydrate metabolism, phytohormone

## Abstract

Winter wheat (*Triticum aestivum* L.) is used as fresh green winter forage worldwide, and its ability to regenerate after mowing determines whether it can be used for forage production; however, the molecular mechanism of regeneration is poorly understood. This study identified long-chain coding and non-coding RNAs in the wheat cultivar “XN9106,” which is cultivated for forage and grain production separately in winter and summer, and analyzed their function during post-mowing regeneration. The results showed that the degradation of carbohydrate plays an important role in regeneration, as demonstrated by decreased carbohydrate content. The increased gene expression of enzymes including β-amylase, β-fructofuranosidase, sucrose synthase, sucrose-6-phosphate synthase, trehalose-6-phosphate synthase, and trehalose-6-phosphate phosphatase in mowed seedlings suggests regeneration is fueled by degraded carbohydrates that provide energy and carbon skeletons for the Krebs cycle and amino acid synthesis. The decreased auxin content relieved the inhibition of cytokinin synthesis, that controls the transition from cell division to cell expansion and stimulates cell expansion and differentiation during the cell expansion phase, and eventually accelerate post-mowing regeneration of seedlings. Additionally, differentially expressed long-chain non-coding RNAs (lncRNAs) might participate in the regulation of gene expression related to carbohydrate metabolism and hormone signal transduction. This study demonstrated the responses of key mRNAs and lncRNAs during post-mowing regeneration of winter wheat and revealed the importance of carbohydrate and hormone during regeneration, providing valuable information for genetic improvement of forage wheat.

## Introduction

Winter wheat (*Triticum aestivum* L.) is one of the most important grain crops worldwide. In many countries, including Argentina, Australia, Pakistan, and the United States, winter wheat is cultivated for dual purposes, both as winter forage and as a summer grain, with high yields and good quality in both roles (Redmon et al., 1995; Arzadun et al., 2006; Mackown et al., 2011; Kim and Anderson, 2015). However, few wheat varieties in China are cultivated for forage production or grazing, which corresponds to the substantial lack of fresh pastures during winter in northern China (Tian et al., 2012). Dual-purpose cultivation of winter wheat has the potential to improve land use efficiency and provide green vegetation protection, compensating for the inability of warm-season forage crops to grow in this region because of the freezing weather conditions during winter (Kim and Anderson, 2015). Compared with other small grain species, winter wheat is the best suited for use as a multipurpose crop because it provides better and more consistent production of pasture, silage, hay, and grain from winter to summer (Royo et al., 1993; Juskiw et al., 2000; Moyer and Coffey, 2000). Therefore, we propose that breeding multipurpose wheat varieties can offer significant economic advantages and fill the need for green winter forage for livestock in northern China.

The ability of wheat seedlings to undergo compensatory regeneration after mowing or grazing is a key factor determining suitability for multipurpose use, as it affects whether the plant can accumulate enough carbohydrates at low winter temperatures to ensure tillering and elongation during the following spring. Studies have shown that excessive grazing and mowing of wheat seedlings for forage can limit grain yield during the following grain harvest season; therefore, it is important to select reliable cultivars and feasible mowing or grazing methods (Arzadun et al., 2006; Tian et al., 2012; Kim and Anderson, 2015). Although there have been many studies on the effects of cultivation conditions and soil nutrients on the multipurpose potential of forage wheat, few have focused on the molecular mechanisms of regeneration after mowing or grazing (Arzadun et al., 2006; Tian et al., 2012). This has greatly limited progress in the genetic improvement of multipurpose wheat varieties.

Carbohydrate metabolism and mobilization provide energy and carbon skeletons for plant growth and development (Hill, 1998; Müller et al., 1999). Plant photosynthesis produces large amounts of carbohydrates and stores them in leaves and stems (Hill, 1998; Fernie et al., 2020). When plants are exposed to various biotic and abiotic stresses, including insect feeding, mechanical damage, drought, and chilling, they cope with the stressful environment by degrading carbohydrates to provide energy and metabolic intermediates (Pommerrenig et al., 2018). Starch and other polysaccharides are degraded into fructose and glucose, which then undergo glycolysis, providing carbon skeletons for other pathways such as amino acid biosynthesis (Pommerrenig et al., 2018). The dynamic changes of carbohydrate are also closely related to plant morphogenesis (Fernandez et al., 2017). Ryegrass and forage wheat must be mowed at the correct height (Arzadun et al., 2006; Turner et al., 2007), as leaving a higher stubble after mowing maintains more carbohydrates, which aids in post-mowing regeneration (Turner et al., 2007). In addition, forage regeneration is a process of leaf cell division and expansion, and leaf growth is controlled by sugar signaling, possibly via the auxin-regulated gene involved in organ size (ARGOS) pathway, which promotes cell division via DNA-binding proteins (Wang and Ruan, 2013). Plant growth is accompanied by cell wall expansion that is a highly sophisticated process, in which carbohydrates are important skeletons and substrates (Verbancic et al., 2018). Furthermore, under stress conditions, plants synthesize various monosaccharides and disaccharides, such as glucose, fructose, galactose, sucrose, and trehalose, which provide a protective environment enabling plant cells to face stress (Pommerrenig et al., 2018). At the same time, the cutting for the forage wheat is also mechanical injury, which can induce changes in plant carbohydrate metabolism (Lukaszuk et al., 2017). In addition, previous study showed that long-chain non-coding RNAs (lncRNAs) might participate in the carbohydrate metabolism. Shi et al. (2020) reported that some lncRNAs is related to the expression regulation of key genes in starch and sucrose metabolisms, which participate in seedling development in rice and Arabidopsis. Although previous studies have shown that carbohydrates play an important role in post-mowing regeneration of wheat, the expression patterns of key genes and the changes in carbohydrate metabolism during this process remain unclear.

Leaf morphogenesis is an intriguing process resulting from the complex interplay of a multitude of regulatory pathways. Phytohormones, including auxins and cytokinins, play a key role in this process (George et al., 2008; Staden et al., 2008; Li et al., 2010; Su et al., 2011). Leaf development originates from the shoot apical meristem (SAM). Cells in the central zone (CZ) of the SAM divide at a relatively low rate and remain undifferentiated, while cells in the peripheral zone (PZ) divide faster and differentiate into various organs such as leaves and axillary nodes (Braybrook and Kuhlemeier, 2010). The slow but continuous division of stem cells in the CZ causes cells to become displaced away from the quiescent center. At a certain position, they lose their stem cell potential and become actively dividing cells. This transition is controlled by the interplay of a regulatory loop involving the homeodomain transcription factor WUSCHEL (WUS) in the PZ and the products of the *CLAVATA* genes (*CLV1*, *CLV2*, and *CLV3*) in the CZ (Kalve et al., 2014). Cytokinin (CK) regulates *WUS* expression via CLV-dependent and CLV-independent mechanisms to promote SAM growth and maintenance (Gordon et al., 2009). In addition, WUS represses the transcription of type A Arabidopsis response regulators (*A-ARRs*), which are negative regulators of CK signaling (Argueso et al., 2010; Kieber and Schaller, 2018). Once progenitor cells are outside the stem cell niche, auxin will decide whether they will contribute to the main axis or differentiate into lateral appendices such as leaf primordia. High concentrations of auxin are accumulated at the beginning of leaf development through regulation by AUX1 and PIN1, which inhibit the activity of KNOX1, limit CK biosynthesis, and promote the formation of leaf primordia in SAM (Su et al., 2011; Guenot et al., 2012; Bar and Ori, 2014). Subsequently, the transformation of the leaf primordium to a mature leaf is controlled by at least six distinct processes: cytoplasmic growth, cell division, endoreduplication, transition between division and expansion, cell expansion, and cell differentiation (Shweta et al., 2014). Most of these processes are tightly controlled by different signaling molecules, including phytohormones. A-type cyclin dependent kinase (CDKA) and D-type cyclin (CYCD) are central to the phase transition of the cell cycle, in which the cell activates DNA duplication (Gaamouche et al., 2010). Plant hormones such as auxin, CK, and gibberellin increase the level of CYCD, thereby activating CDKA (Inzé and De Veylder, 2006; Perrot-Rechenmann, 2010). It has been observed that the leaf cells of Arabidopsis go from mitosis to endoreduplication as the auxin concentration decreases (Ishida et al., 2010). Auxin plays an important role in the transition between cell division and expansion, as it induces the expression of the *ARGOS* gene (Hu et al., 2003). However, auxin does not always promote cell expansion, as its concentration has also been observed to decrease during leaf expansion (Braun et al., 2008), indicating a more complex dose–response relationship. The function of CK is likewise complicated. During cell division, CK is needed to maintain cell proliferation by blocking the transition to cell expansion and the onset of photosynthesis, while during the cell expansion phase, cytokinin stimulates cell expansion and differentiation (Skalák et al., 2019). During the post-mowing regeneration of winter wheat, the reconstruction of leaf morphology is dramatic, and phytohormones play a crucial role in this process. Application of exogenous cytokinins has been reported to promote post-mowing regeneration of barley during maturity (Christiansen et al., 1995). Further, lncRNAs play an important role in the growth and development of plants (Yamada, 2017; Matsui and Seki, 2019). Zubko and Meyer (2007) identified a natural antisense lncRNA that plays an important role in regulating the biosynthesis of cytokinins. Zhu et al. (2015) reported that lncRNAs participate in the regulation of auxin concentrations during the development phase of fruit ripening (2015). Therefore, studying changes in the expression of genes and lncRNAs related to phytohormone biosynthesis and signal transduction will help to elucidate the role of phytohormones in the post-mowing regeneration of forage winter wheat.

RNA plays an important role in plant growth and development, whether as mRNA or as a regulator such as microRNA, lncRNA, and so on. mRNA is a direct product of gene transcription, and plays an important role in the post-mowing regeneration of forage wheat. Study has shown that microRNA is involved in the post-mowing regeneration of wheat and plays an important role in the regulation of gene expression (Cui et al., 2020). LncRNAs have also been reported to be involved in the development of shoot meristems in Populus (Liu et al., 2019) and photomorphogenesis in Arabidopsis (Wang et al., 2014). Therefore, we infer that mRNAs and lncRNAs likely have important functions in the post-mowing regeneration of forage winter wheat. However, few studies have examined the role of lncRNAs in the post-mowing regeneration of forage wheat. Additionally, no previous studies have sequenced mRNAs and lncRNAs in an attempt to reveal the molecular mechanism of compensatory regeneration in winter wheat seedlings after mowing. To reveal the molecular responses of mRNAs and lncRNAs during post-mowing regeneration of winter wheat, wheat seedlings were mowed, and the stubble (including leaf sheath and young leaves in it) was harvested for transcriptional and physiological assays within 72 h after mowing. The functions of differentially expressed mRNAs and lncRNAs were annotated using orthologous alignment, and their interactions were predicted. This study showed the responses of mRNAs and lncRNAs during post-mowing regeneration of winter wheat, and suggested that carbohydrate and hormones are involved in the post-mowing regeneration of wheat.

## Materials and Methods

### Materials and Growth Rate Determination

**FIGURE 1:**
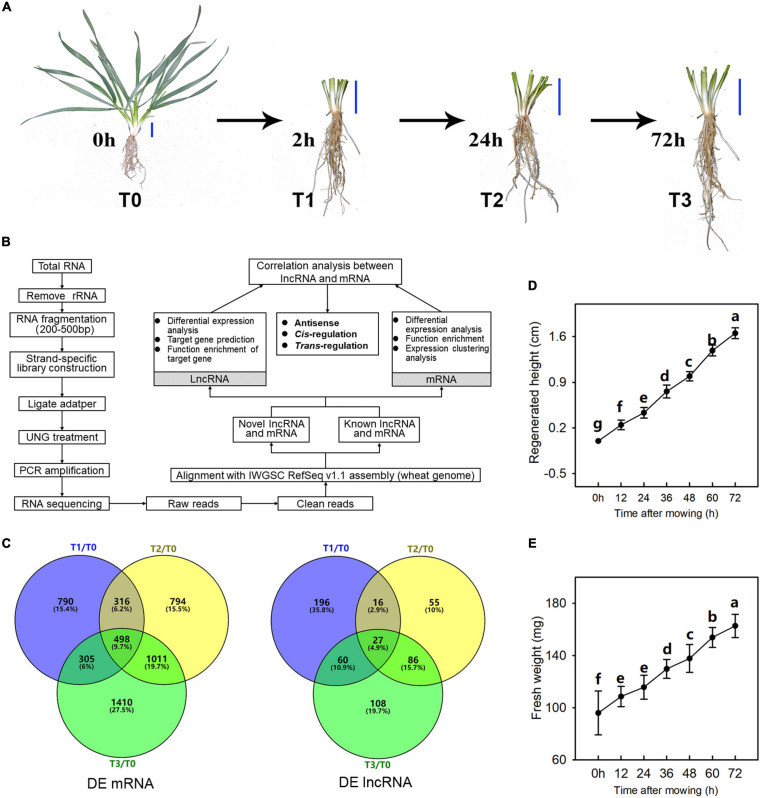
Regeneration process of wheat seedlings following mowing. **(A)** Regeneration process of wheat seedlings and sampling time (T0, T1, T2, and T3 means the sample was harvested at 0, 2, 24, and 72 h after mowing, respectively.). The blue bar represents a three-centimeter reference growth scale. **(B)** RNA isolation and bioinformatic analysis. Three biological replicates were used for this analysis. **(C)** Venn diagram of differentially expressed mRNAs (DE mRNA) and lncRNAs (DE lncRNA). **(D,E)** The wheat seedlings were harvested every 12 h within 72 h after mowing. The height of regenerated young leaves was measured, and the fresh weight of the aboveground parts of the main tiller of wheat seedlings was determined. Data are the mean values of 15 independent replicates (*n* = 15), and error bars show the standard error. Duncan’s multiple-comparison test was used for ANOVA, and different lowercase letters indicate a significant difference at the level of *P* < 0.05.

The winter wheat strain used in this study (*T. aestivum* L. “XN9106”) was selected because of its large biomass and high regeneration capacity after mowing, as observed in our previous study (Liu et al., 2018). Wheat seedlings were planted in the experimental fields of Northwest Agriculture and Forestry University, Yangling, Shaanxi, China (34°16′56.24″N, 108°4′27.95″E). The experiment was carried out in an arid pond (0.4 × 4 × 5 m). The soil is a mixture of farmland topsoil/sand/grass peat (1:1:2, v:v:v) (pH, 7.62; organic matter, 45.16 g/kg; available N, P, and K, 53.11, 30.15, 71.77 g/kg; maximum field capacity, FC, 20.32%). The irrigation of the experimental fields is controlled manually and soil moisture content maintained at 80% of FC. The sowing density of wheat is 5 × 15 cm and sowing date is September 20, 2018. The average temperatures in September, October and November are 20°C, 15°C, and 7°C. A single factor completely random design was conducted in this experiment. When the seedlings had 4–5 tillers, the samples were firstly harvested. The 2-cm-tall stem (including leaf sheath and young leaves in it) of at least 15 seedlings, measured from the base of the stem, were harvest at 9 am by removing the outermost sheath and was marked as T0 (Figure 1A). While obtaining the T0 sample, parts above 3 cm of all other seedlings were also mowed at 9 am. T1, T2, and T3 sample were harvested at 2, 24, and 72 h after mowing, and the method was the same as T1 samples (Figure 1A). The regenerated fresh weight and height of seedlings were determined every 12 h after mowing, with 15 plants measured. Duncan’s multiple-range test (*P* < 0.05) were used to detect differences for weight and height determination.

### Sugar and Starch Assays

Determination of sugar and starch content was performed according to the method described by Mccready et al. (1950), using five biological replicates. Duncan’s multiple-range test (*P* < 0.05) were used to detect differences among different samples. Total sugar and reducing sugars were extracted from 0.2 g of the sample with 10 mL of 80% ethanol. The extract was centrifuged at 5000 × *g* for 15 min. The supernatant was used to determine the total sugar and reducing sugar content. The starch in the precipitate was first gelatinized with 2 mL of distilled water at 100°C for 15 min and then dissolved in 2 mL of 9.6 M perchloric acid. The precipitate was repeatedly extracted with 2 mL of perchloric acid and washed twice with distilled water. The extract was centrifuged at 5000 × *g* for 15 min, after which all the supernatants were combined and the total volume was increased to 20 mL using distilled water. Reducing sugar content was determined using a 4-mL reaction system including 2 mL of 1% (m/v) 3,5-dinitrosalicylic acid and 2 mL of the supernatant. Total sugar and starch were determined in a 4 mL reaction system including 3 mL of 0.2% (m/v) anthrone-sulfuric acid solution and 1 mL of the extract. The sugar and starch contents were calculated using a standard curve.

### Measurement of Auxins and *cis*-Zeatin

Acetonitrile and methanol of the proper grade for high-performance liquid chromatography (HPLC) were purchased from Merck (Darmstadt, Germany). Standards were purchased from Olchemim Ltd. (Olomouc, Czechia) and Sigma (St. Louis, MO, United States). Acetic acid was sourced from Sinopharm Chemical Reagents (Shanghai, China). Fifteen seedlings were harvested for each of the three biological replicates used for quantification. Fresh plant materials were harvested, weighed, snap-frozen in liquid nitrogen, and stored at −80°C. Endogenous phytohormone determination was performed according to Pan et al. (2008). Plant materials (500 mg fresh weight) were ground into a powder and extracted using methanol (80%, v/v) at 4°C. The extract was centrifuged at 12,000 × *g* at 4°C for 15 min. The supernatant was collected and dried under a nitrogen gas stream and subsequently redissolved in methanol (30%, v/v). The solution was centrifuged, and the supernatant was collected for liquid chromatography–mass spectrometry (LC-MS) analysis.

Sample extracts were analyzed using an LC-electrospray ionization (ESI)-MS/MS system (HPLC, Shim-pack UFLC Shimadzu CBM30A System, Shimadzu, Japan; MS, Applied Biosystems 6500 Triple Quadrupole, Thermo Fisher Scientific, Waltham, MA, United States). The analytical conditions were as follows: HPLC: column, Waters ACQUITY UPLC HSS T3 C18 (1.8 μm, 2.1 mm × 100 mm); solvent system, water (0.04% acetic acid): acetonitrile (0.04% acetic acid); gradient program, 95:5% (v/v) at 0 min, 5:95% at 11.0 min, 5:95% at 12.0 min, 95:5% at 12.1 min, 95:5% at 15.0 min; flow rate, 0.35 mL/min; temperature, 40°C; and injection volume: 5 μL. The effluent was alternatively connected to an ESI-triple quadrupole–linear ion trap (Q TRAP)-MS system.

The effluent was analyzed using the API 6500 Q TRAP LC/MS/MS system, equipped with an ESI turbo ion-spray interface, operating in positive ion mode and controlled using Analyst 1.6 software (AB Sciex, Framingham, MA, United States). ESI source operation parameters were as follows: ion source, turbo spray; source temperature, 500°C; ion spray voltage (IS), 5500 V; curtain gas (CUR), 35.0 psi; and collision gas (CAD), medium. Declustering potential (DP) and collision energy (CE) were set for individual multiple reactions monitoring (MRM) transitions, followed by further DP and CE optimization. A specific set of MRM transitions were monitored for each period according to the plant hormones eluted within this period. Endogenous hormone levels were quantified according to a standard curve (Supplementary Figure 1). There were three biological replicates and total 12 samples. Duncan’s multiple-range test (*P* < 0.05) were used to detect the differences among different samples.

### RNA Extraction, Library Construction, and Sequencing

The RNA sequencing procedure is shown in Figure 1, and three biological replicates were used for each treatment. Total RNA of 12 samples was extracted from wheat seedlings at the aforementioned time points using the TRIzol reagent (Invitrogen, Carlsbad, CA, United States). The quantity, purity, and integrity of the resulting RNA were confirmed using NanoDrop One (Thermo Scientific, United States) and Agilent 2100 (Agilent Technologies, United States) instruments. Only when the RNA integrity value of the sample was ≥8 were RNAs used for the next test.

After the quality evaluation, rRNAs were removed; mRNAs and non-coding RNAs (ncRNAs) were retained using the Ribo-Zero rRNA Removal Kit (Illumina, San Diego, CA, United States). The enriched mRNAs and ncRNAs were fragmented into short fragments using a fragmentation buffer and were reverse-transcribed into cDNA with random primers. The cDNAs were used for library construction. The protocols for library construction and sequencing followed the standard procedures provided with the NEBNextUltra^TM^ RNA Library Prep Kit (Illumina). Sequencing was completed using the Illumina HiSeq^TM^ 4000 at the Gene *De novo* Biotechnology Company (Guangzhou, China).

### Identification and Characterization of mRNAs and LncRNAs

All raw reads were processed using Perl scripts, in which high-quality clean reads from 12 samples were obtained by removing reads containing adapters, more than 10% unknown nucleotides, and low-quality bases. The clean reads were aligned to the wheat ribosome RNA database^1^ by using Bowtie 2 (2.2.8) (Langmead and Salzberg, 2012), and reads mapped to ribosome RNA database were removed. The filtered reads were then mapped to the wheat reference genome (IWGSC RefSeq v1.1)^2^ using TopHat 2 (2.2.2) (Kim et al., 2013).

The transcriptome of each sample were assembled independently using Cufflinks (2.2.1) together with TopHat 2 to form a consensus transcriptome assembly (Cole et al., 2012). All reconstructed transcripts were aligned to the wheat reference genome (IWGSC_RefSeq_v1.0) using Cuffcompare to identify mRNAs. These mRNAs were then aligned to the Nr, Kyoto Encyclopedia of Genes and Genomes (KEGG), and Gene Ontology (GO) databases to obtain protein functional annotation. In order to further analyze the gene function, all identified mRNAs were aligned with Arabidopsis and rice genes using blastn (E ≤ 10^–5^). The gene of Arabidopsis and rice with the highest score is regarded as the homologous gene of wheat. The gene ID and their GO and KEGG annotation are listed in Supplementary Table 3.

For lncRNA prediction, the transcripts above were first filtrated according to the position of the reference wheat genome and two filtrated standards (sequence length ≥200 bp and the number of exons ≥1). A known and a novel transcriptome was obtained from that assembled transcriptome. To identify lncRNAs, SwissProt databases (BlaxtX, E ≤ 10^–5^), the CNCI (2.0) and the Coding Potential Calculator (CPC)^3^ were used to assess the protein-coding potential of novel transcripts using default parameters (Lei et al., 2007; Zhang et al., 2013). Sequences lacking both protein-coding potential results and protein annotation results were identified as lncRNAs.

### Differential Expression Analysis and Trend Analysis

Transcript abundance was quantified by RSEM software, and transcript levels were normalized according to the fragments per kilobase of transcript per million mapped reads (FPKM) (Bo and Dewey, 2011). EdgeR package^4^ was used to identify differentially expressed genes (DEGs) between pairs of samples (| fold change| ≥ 2 and false discovery rate <0.05). Trend analysis of mRNAs was performed using Short Time-series Expression Miner software (Ernst and Bar-Joseph, 2006) on the OmicShare tools platform^5^. For GO and KEGG enrichment, the calculated *P*-values were subjected to FDR correction, using FDR ≤ 0.05 as a threshold. GO terms or KEGG pathways meeting this condition were defined as significantly enriched in differentially expressed mRNAs.

### Interaction Analysis Between LncRNAs and mRNAs

To reveal interactions between antisense lncRNAs and mRNAs, RNAplex software^6^ was used to predict the complementary correlation of antisense lncRNAs and mRNAs (Hakim and Hofacker, 2008). The program contains the ViennaRNA package, and the prediction of best base pairing was based on the calculation of minimum free energy through a thermodynamic structure. LncRNAs less than 100 kb upstream of a gene were identified as *cis*-regulators. The *cis* target mRNAs were identified as interacting with related lncRNAs. The correlation of expression between lncRNAs and mRNAs was analyzed to identify target genes of lncRNAs. The Pearson correlation coefficient was used, and protein-coding genes with an absolute correlation greater than 0.9 were identified as being correlated. After an interaction between lncRNA and mRNA was identified, the interaction was illustrated with Cytoscape 3.4.0 (Shannon, 2003). The expression levels of interacting lncRNAs and mRNAs are listed separately to show the effect of their interaction.

### Relative qRT-PCR Analysis

To verify the expression of some genes, real-time fluorescence relative quantitative PCR was used. Four biological replicates were used to detect the expression level of target genes, and 10 plants were used for each biological replicate. Duncan’s multiple-range test (*n* = 4, *P* < 0.05) were used to detect differences among different samples. For RNA reverse transcription, 2 μg of total RNA was reverse-transcribed using the PrimeScript^TM^ RT Reagent Kit (TaKaRa BIO, Japan). The gene-specific primers were designed using Primer Premier 6.0 and are shown in Supplementary Table 1; quantitative real-time polymerase chain reaction (qRT-PCR) was performed in a 20-μL volume according to the manufacturer’s instructions (SYBR^®^ Advantage^®^ qPCR Premix, TaKaRa BIO). The two-step PCR method was used under the following conditions: pre-denaturation at 95°C for 5 min, 40 cycles of 95°C for 15 s, and 60°C for 30 s. For gene expression normalization, *actin (gene ID: AB181991)* was used as an internal reference.

### Data Visualization

Microsoft Excel 2016 (Microsoft Corp., Redmond, WA, United States) and SigmaPlot 12.5 (Systat Software Inc., San Jose, CA, United States) were used for data arrangement and visualization. The software IBM SPSS Statistics 19.0 (IBM Corp., Armonk, NY, United States) was used for analysis. The interaction diagram for lncRNAs and mRNAs was created with Cytoscape 3.4.1^7^.

## Results

### Determination of Growth Parameters and Physiological Parameters

In this study, we assessed the post-mowing regeneration of wheat seedlings and the growth parameters of regenerated seedlings are shown in Figure 1. The samples harvested at 0, 2, 24, and 72 h after mowing were labeled as T0, T1, T2, and T3, respectively. After mowing, young wheat leaves grew longer than the leaf sheaths of the old leaves. The height of the regenerated wheat leaves reached more than 1.5 cm (Figure 1D). The fresh weight of the regenerated wheat leaves also increased (Figure 1E). To observe the post-mowing regeneration of wheat directly, an indoor simulation of post-mowing regeneration was conducted in a growth chamber at a temperature of 15/20°C under a 10/14 h dark/light cycle. Visible regeneration occurred 2 h after mowing, and the length of regenerated young leaves was up to 3 cm within 72 h after mowing (Supplementary Video 1).

**FIGURE 2:**
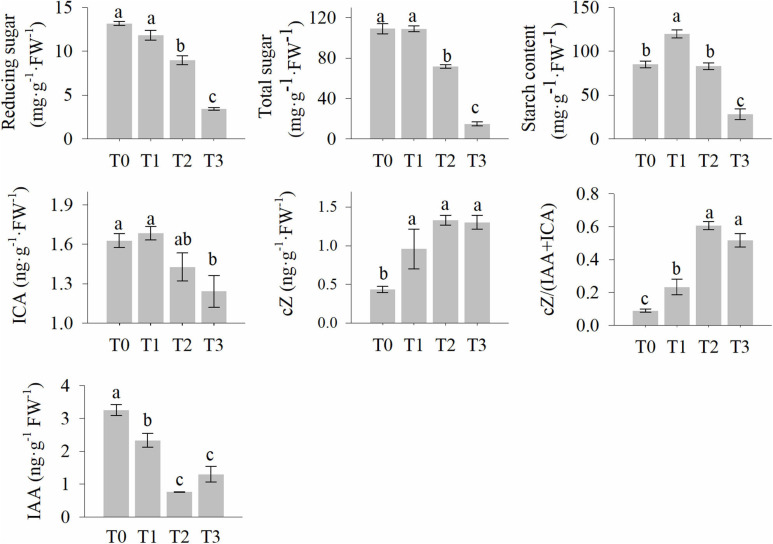
The levels of starch, soluble sugar, and phytohormone in wheat stubble after mowing. Data are the mean values (all measurement were conducted at least three biological replicates), and error bars show the standard error. Duncan’s multiple-comparison test was used for ANOVA, and different lowercase letters indicate a significant difference at the level of *P* < 0.05. T0, T1, T2, and T3 represent the samples harvested at 0, 2, 24, and 72 h after mowing.

The reducing sugar, total sugar, and starch content of the wheat stubble are shown in Figure 2. They decreased significantly within 72 h after mowing (Figure 2). However, the starch content increased at 2 h (Figure 2). This suggests that carbohydrates are involved in the post-mowing regeneration of seedlings. Levels of auxins and cytokinins were also determined and are shown in Figure 2. The indole-3-acetic acid (IAA) and indole-3-carboxaldehyde (ICA) content of seedling stubble decreased significantly within 72 h after mowing, while the cZ (*cis*-zeatin) content in the seedling stubble increased significantly at 24 and 72 h after mowing (Figure 2). This indicates the important roles of auxins and cytokinins in the post-mowing regeneration of wheat seedlings.

### Expression Profiles of mRNAs and LncRNAs

To study the expression profiles of long-chain coding and non-coding RNAs during wheat regeneration, total RNA was harvested from wheat stubble. After RNA sequencing, filtering, and reconstruction, mRNAs and lncRNAs were identified. All identified and predicted known and new mRNAs and lncRNAs in the samples are shown in Table 1. In all samples, 92551 known mRNAs and 225 known lncRNAs were identified, and 88599 new mRNAs and 16477 new lncRNAs were predicted.

**TABLE 1:** The identified mRNAs and lncRNAs in all samples.

Sample Name	The number of			The number of		
	identified mRNAs			identified lncRNAs		
		
	Known	New	Total	Known	New	Total
T0-1	63005	71836	134841	34	11834	11868
T0-2	64639	74000	138639	43	12685	12728
T0-3	65692	75139	140831	52	12778	12830
T1-1	65763	74279	140042	40	12453	12493
T1-2	64326	73014	137340	26	12274	12300
T1-3	64832	73765	138597	39	12445	12484
T2-1	63912	72144	136056	29	11895	11924
T2-2	63454	70915	134369	23	11575	11598
T2-3	68093	75625	143718	48	12863	12911
T3-1	64661	73167	137828	40	12010	12050
T3-2	67189	76154	143343	44	12924	12968
T3-3	66554	76024	142578	48	12812	12860
Total	92551	88599	181150	225	16477	16702

**FIGURE 3:**
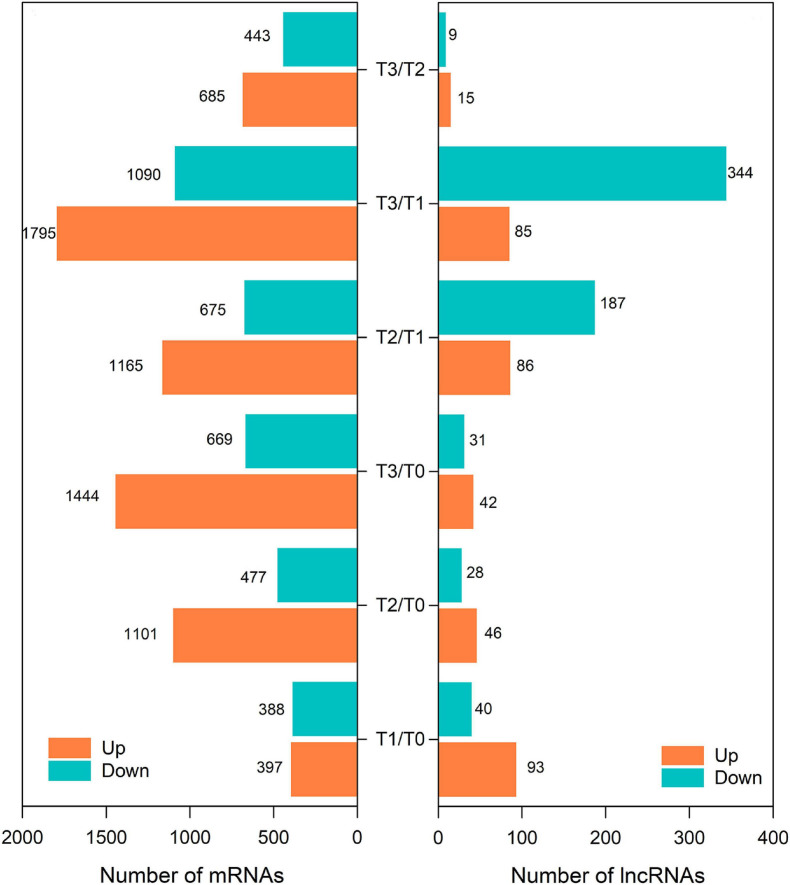
The number of differentially expressed mRNAs and lncRNAs in wheat seedlings after mowing.

The differentially expressed (DE) mRNAs and lncRNAs between different experimental groups are shown in Figures 1C, 3, respectively. There were 5882 mRNAs and 672 lncRNAs that were differentially expressed between the two experimental groups (Supplementary Tables 2, 3). DE mRNAs in wheat seedlings gradually increased within 72 h after mowing and were mainly concentrated in the T2 and T3 groups (Figure 3). There were more DE mRNAs in the T3/T1 and T3/T0 pairings than in the T2/T1 and T1/T0 pairings. The number of DE mRNAs in the T1/T0 pairing was the lowest among all the comparison groups. The greatest numbers of DE lncRNAs were found in the T3/T1 and T2/T1 pairings, which was similar to the case for DE mRNAs (Figure 3). Unlike mRNAs, the number of DE lncRNAs in the T1/T0 pairing was more than the number in T2/T0, T3/T0, and T3/T2. Additionally, the number of DE lncRNAs in T3/T0 was the lowest among all comparison groups. This indicates that expression profiles differed between DE mRNAs and DE lncRNAs.

### KEGG Enrichment and Expression Trend Cluster Analysis of DE mRNAs

**FIGURE 4:**
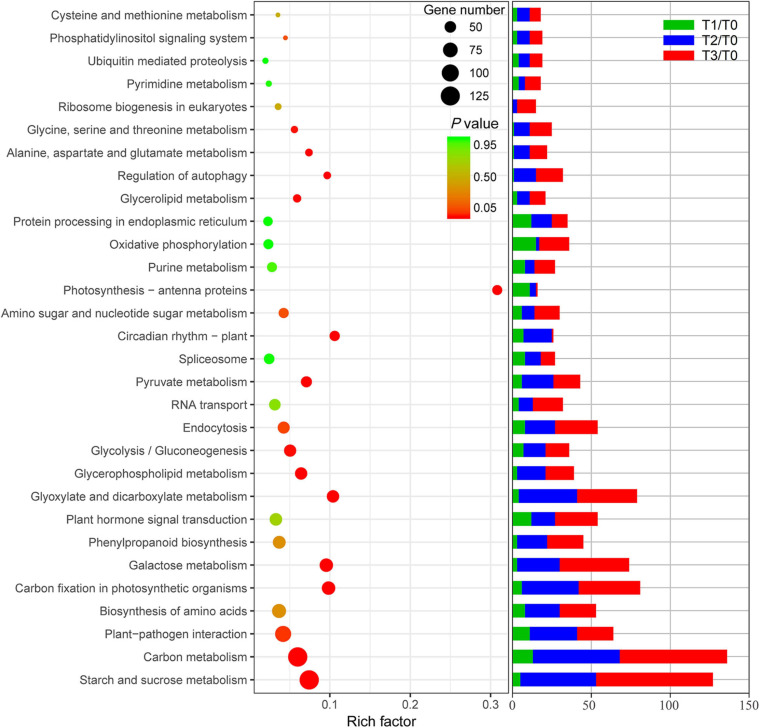
KEGG enrichment of differentially expressed mRNAs and mRNAs of different pathways in different comparison groups.

All DE mRNAs were annotated in KEGG database and are shown in Supplementary Table 3. The result of KEGG enrichment is shown in Figure 4. In the KEGG enrichment analysis, more than 50 mRNAs were enriched, including some involved in starch and sucrose metabolism, carbon metabolism, plant-pathogen interaction, carbon fixation in photosynthetic organisms, amino acid biosynthesis, galactose metabolism, phenylpropanoid biosynthesis, plant hormone signal transduction, glyoxylate and dicarboxylate metabolism, glycerol-phospholipid metabolism, glycolysis/gluconeogenesis, endocytosis, and RNA transport. Furthermore, most of the DE mRNAs in T3/T0, T2/T0, and T1/T0 were concentrated in these pathways. Enriched DE mRNAs in T1/T0 were involved in oxidative phosphorylation, protein processing in the endoplasmic reticulum, carbon metabolism, plant-pathogen interaction, plant hormone signal transduction, and photosynthesis-antenna proteins. These pathways are more focused on signal transmission and light morphogenesis. The enriched DE mRNAs in T2/T0 and T3/T0 were mainly involved in starch and sucrose metabolism, carbon metabolism, plant-pathogen interaction, carbon fixation in photosynthetic organisms, amino acid biosynthesis, galactose metabolism, plant hormone signal transduction, glyoxylate and dicarboxylate metabolism, and endocytosis. These pathways are more focused on the synthesis and degradation of carbohydrates and amino acids.

To further illuminate the expression profile of mRNAs at different regeneration stages, a trend clustering analysis was performed. A total of 26 profiles of mRNAs in the regeneration stage are shown in Supplementary Figure 2 and Supplementary Table 4. Profiles 7 and 18 were classified as class I (Supplementary Figure 2A). The expression of mRNAs in class I changed at 2 h but not at 24 h and 72 h after mowing. KEGG enrichment of these mRNAs showed that most were involved in plant circadian rhythms, oxidative phosphorylation, ubiquitin-mediated proteolysis, carbon metabolism, and purine metabolism (Supplementary Figure 2B). Profiles 9–16 were classified as class II, and the expression of mRNAs in class II did not change at 2 h but did not change at 24 or 72 h after mowing (Supplementary Figure 2A). These mRNAs were concentrated in carbon metabolism, starch and sucrose metabolism, galactose metabolism, amino sugar and nucleotide sugar metabolism, and glycolysis/gluconeogenesis (Supplementary Figure 2C). Profiles 0–4 and 21–24 were classified as class III, and the expression of mRNAs in this class was altered at 2, 24, and 72 h after mowing (Supplementary Figure 2A). KEGG enrichment showed that most enriched mRNAs were involved in glycerol-phospholipid metabolism, carbon fixation in photosynthetic organisms, endocytosis, carbon metabolism, and plant-pathogen interaction (Supplementary Figure 2D).

### Carbohydrate Degradation and Mobilization

**FIGURE 5:**
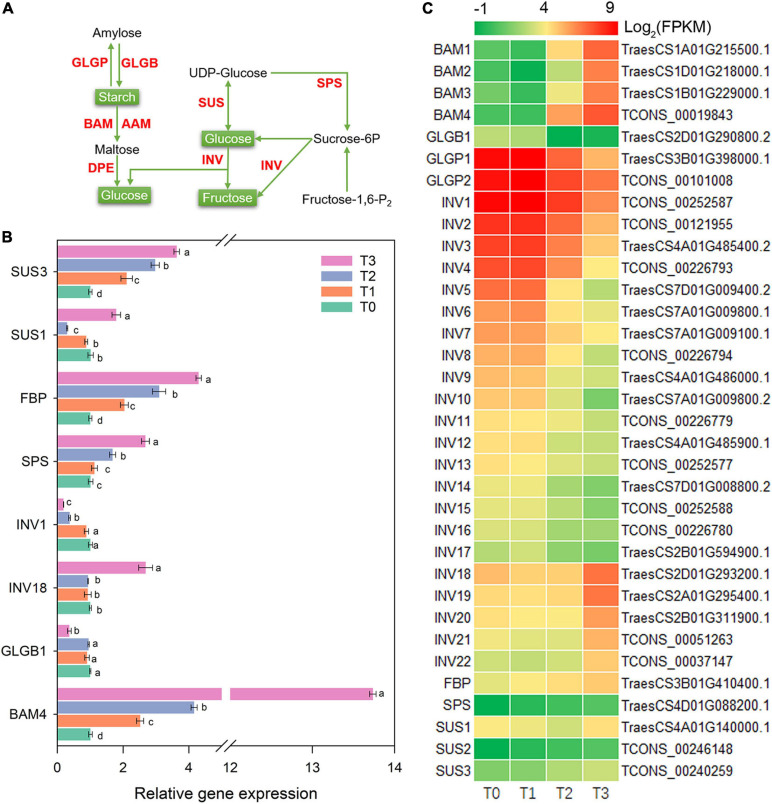
Genes involved in starch and sucrose metabolism. AAM, α-amylase; BAM, β-amylase; DPE, 4-α-glucanotransferase; GLGB, 4-α-glucan branching enzyme; GLGP, amylophosphorylase; INV, β-fructofuranosidase; SPS, sucrose-6-phosphate synthase; FBP, fructose-1,6-bisphosphatase; SUS, sucrose synthase. **(A)** Pathway map of starch and sucrose metabolism. **(B)** Verification of the expression of some genes in the pathway. Data are the mean values of four independent replicates (*n* = 4), and error bars show the standard error. Duncan’s multiple-comparison test was used for ANOVA, and different lowercase letters indicate a significant difference at the level of *P* < 0.05. **(C)** Heatmap of differentially expressed mRNAs in this pathway. T0, T1, T2, and T3 means the sample was harvested at 0, 2, 24, and 72 h after mowing, respectively.

During the post-mowing regeneration of wheat seedlings, the gene expression related to starch and sucrose metabolism was altered (Figure 5). Gene expression of *BAM1–4*, encoding BAM (β-amylase) in starch degradation, significantly increased (Figure 5C). The expression of *BAM4* in relative qRT-PCR verified above result and is shown in Figure 5B. INV (β-Fructofuranosidase) is a unidirectional enzyme for sucrose degradation and is encoded by *INV1-22*. In this study, the expression of *INV1–17* was decreased after mowing, while the expression of *INV18–22* was increased (Figure 5C). The expression of *INV1* and *INV18* in RNA-seq was also verified by qRT-PCR in Figure 5B. Additionally, gene expression of two other key enzymes, sucrose-6-phosphate synthase (SPS) and sucrose synthase (SUS), was also altered (Figure 5C). The expression of *SPS* and *SUS3* increased significantly within 72 h after mowing according to qRT-PCR and is shown in Figure 5B.

**FIGURE 6:**
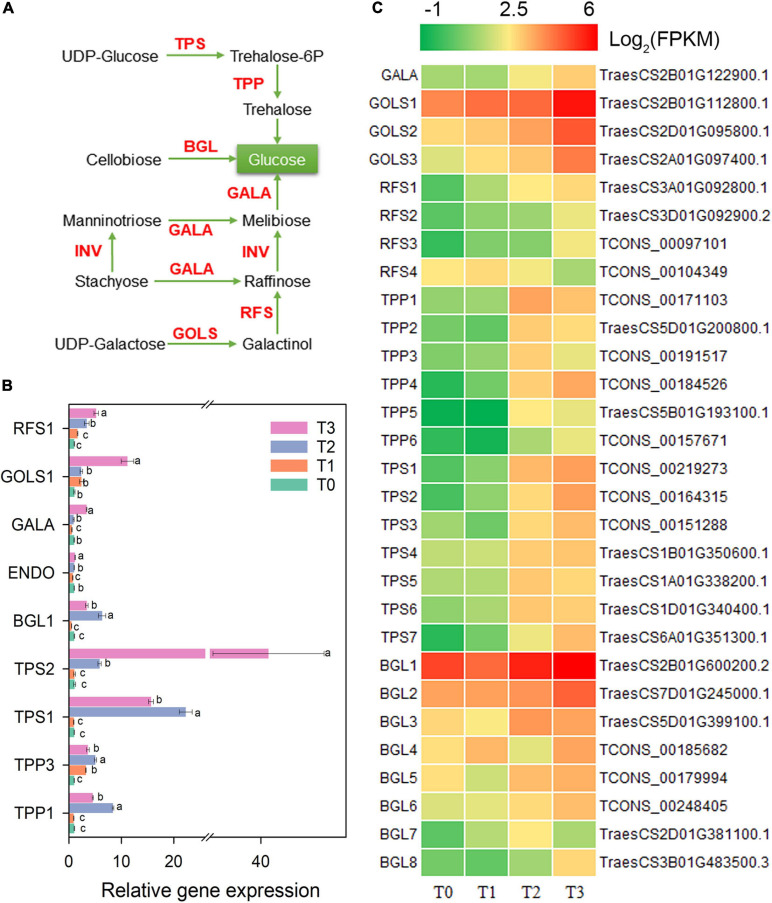
Genes involved in the metabolism of trehalose, cellulose, melibiose, raffinose, and galactose. GALA, α-galactosidase; GOLS, inositol 3-α-galactosyltransferase; RFS, raffinose synthase; TPP, trehalose-6-phosphate phosphatase; TPS, trehalose-6-phosphate synthase; BGL, β-glucosidase. **(A)** Pathway map of trehalose, cellulose, melibiose, raffinose, and galactose. **(B)** Verification of the expression of some genes in the pathway. Data are the mean values of four independent replicates (*n* = 4), and error bars show the standard error. Duncan’s multiple-comparison test was used for ANOVA, and different lowercase letters indicate a significant difference at the level of *P* < 0.05. **(C)** Heatmap of differentially expressed mRNAs in this pathway. T0, T1, T2, and T3 means the sample was harvested at 0, 2, 24, and 72 h after mowing, respectively.

The metabolism of trehalose, cellulose, melibiose, stachyose, raffinose, and galactose might be changed during post-mowing regeneration of wheat seedlings according to the changed expression of related genes (Figure 6). Within 72 h after mowing, the gene expression of two key enzymes for trehalose synthesis, trehalose-6-phosphate phosphatase (TPP) and trehalose-6-phosphate synthase (TPS), increased significantly and is shown in Figure 6C. The expression of *TPP1*, *TPP3*, *TPS1*, and *TPS2* also increased by qRT-PCR in Figure 6B. After mowing, the expression of *BGL1-8*, which encoding BGL (β-glucosidase, a key enzyme for cellobiose degradation), was significantly increased (Figure 6C). The expression of *BGL1* also increased within 72 after mowing according to qRT-PCR in Figure 6B. Furthermore, the expression of genes encoding α-galactosidase (GALA), inositol 3-α-galactosyltransferase (GOLS), and raffinose synthase (RFS) was affected during the regeneration of seedlings (Figure 6C). The expression of *RFS1* and *GOLS1* increased significantly within 72 h after mowing (Figure 6B).

### Glycolysis, Gluconeogenesis, and Krebs Cycle

**FIGURE 7:**
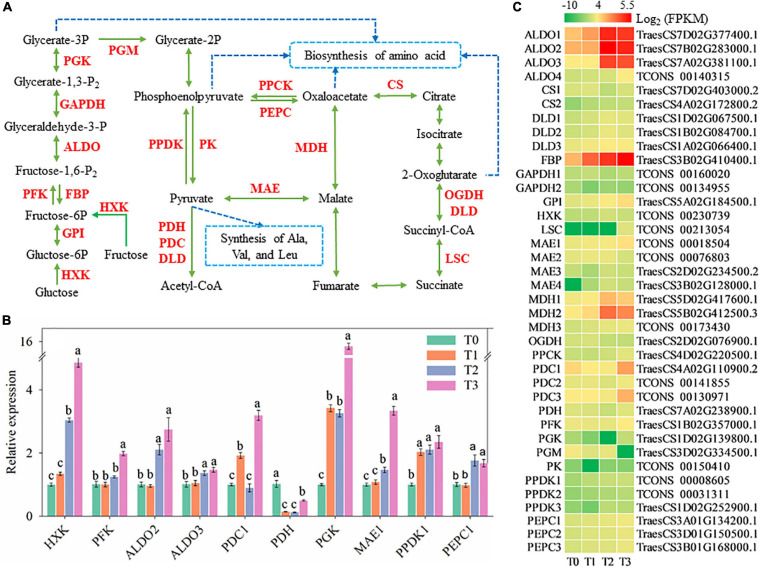
Genes involved in glycolysis and gluconeogenesis. HXK, hexokinase; GPI, glucose-6-phosphate isomerase; PFK, phosphofructokinase; FBP, fructose-1,6-bisphosphatase; ALDO, fructose-bisphosphate aldolase; GAPDH, glyceraldehyde-3-phosphate dehydrogenase; PGK, phosphoglycerate kinase; PGM, phosphoglycerate mutase; PPDK, pyruvate-phosphate dikinase; PK, pyruvate kinase; PPCK, phosphoenolpyruvate carboxykinase; PEPC, phosphoenolpyruvate carboxylase; MAE, malic enzyme; MDH, malate dehydrogenase; PDC, pyruvate decarboxylase; PDH, pyruvate dehydrogenase; DLD, dihydrolipoamide dehydrogenase; CS, citrate synthase; OGDH, 2-oxoglutarate dehydrogenase; LSC, succinyl-CoA synthetase. **(A)** Pathway map of glycolysis and gluconeogenesis. **(B)** Verification of the expression of some genes in the pathway. Data are the mean values of four independent replicates (*n* = 4), and error bars show the standard error. Duncan’s multiple-comparison test was used for ANOVA, and different lowercase letters indicate a significant difference at the level of *P* < 0.05. **(C)** Heatmap of differentially expressed mRNAs in this pathway. T0, T1, T2, and T3 means the sample was harvested at 0, 2, 24, and 72 h after mowing, respectively.

Glycolysis and gluconeogenesis are important branches of carbohydrate metabolism and might be affected at 72 h after mowing (Figure 7). Expression of genes encoding hexokinase (HXK), glucose-6-phosphate isomerase (GPI), phosphofructokinase (PFK), fructose-1,6-bisphosphatase (FBP), fructose-bisphosphate aldolase (ALDO), glyceraldehyde-3-phosphate dehydrogenase (GAPDH), phosphoglycerate kinase (PGK), pyruvate-phosphate dikinase (PPDK), and pyruvate decarboxylase (PDC) was significantly increased (Figure 7C). The expression of *FBP*, *HXK*, *PFK*, *ALDO2*–*3*, *PGK*, and *PPDK1* increased within 72 h after mowing according to qRT-PCR and is shown in Figures 5B, 7B. Their expression is generally consistent with the experimental results of RNA-seq. As a result of the enhanced expression of these enzymes, glucose, and fructose might be degraded into pyruvate and converted to other carbohydrates. Furthermore, pyruvate might be further degraded and converted into a skeleton of other carbohydrates. In addition, the expression of some key genes related to the Krebs cycle was changed during post-mowing regeneration (Figure 7C), which encoding enzymes including phosphoenolpyruvate carboxykinase (PPCK), phosphoenolpyruvate carboxylase (PEPC), malate dehydrogenase (MDH), malic enzyme (MAE), citrate synthase (CS), 2-oxoglutarate dehydrogenase (OGDH), dihydrolipoamide dehydrogenase (DLD), and succinyl-CoA synthetase (LSC). The expression of *PDC1*, *PDH*, and *PEPC1* in post-mowing wheat according to qRT-PCR is also shown in Figure 7B and is also generally consistent with the result of RNA-seq in Figure 7C.

### Carbon Fixation

**FIGURE 8:**
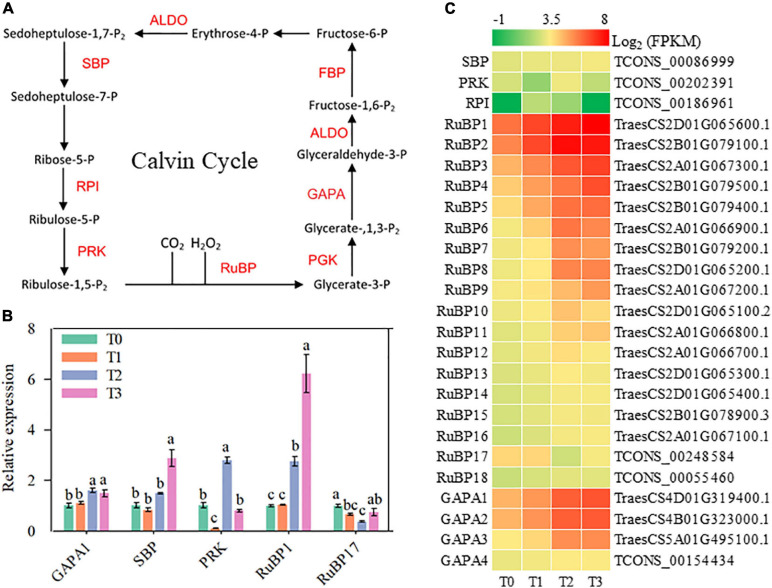
Genes involved in carbon fixation and malate metabolism. ALDO, fructose-bisphosphate aldolase; FBP, fructose-1,6-bisphosphatase; GAPA, glyceraldehyde-3-phosphate dehydrogenase; PGK, phosphoglycerate kinase; RuBP, ribulose bisphosphate carboxylase; PRK, phosphoribulokinase; RPI, ribose-5-phosphate isomerase; SBP, sedoheptulose-1,7-bisphosphatase. **(A)** Pathway map of carbon fixation and malate metabolism. **(B)** Verification of the expression of some genes in the pathway. Data are the mean values of four independent replicates (*n* = 4), and error bars show the standard error. Duncan’s multiple-comparison test was used for ANOVA, and different lowercase letters indicate a significant difference at the level of *P* < 0.05. **(C)** Heatmap of differentially expressed mRNAs in this pathway. T0, T1, T2, and T3 means the sample was harvested at 0, 2, 24, and 72 h after mowing, respectively.

The Calvin cycle is the main pathway of carbon fixation in C3 plants. During post-mowing regeneration of wheat seedlings, the activity of the Calvin cycle might be enhanced, as suggested by the increased expression of some key genes, including *ALDO1-4*, *SBP* (encoding sedoheptulose-1,7-bisphosphatase), *RuBP1-18* (encoding ribulose bisphosphate carboxylases), and *GAPA1-4* (encoding glyceraldehyde-3-phosphate dehydrogenases) in Figures 7C, 8C. Furthermore, the expression of *GAPA1*, *SBP*, *ALDO2–3*, *RuBP1*, and *PGK* increased during post-mowing regeneration of wheat seedlings, according to qRT-PCR (Figures 7B, 8B).

### Plant Hormone Biosynthesis and Signal Transduction

**FIGURE 9:**
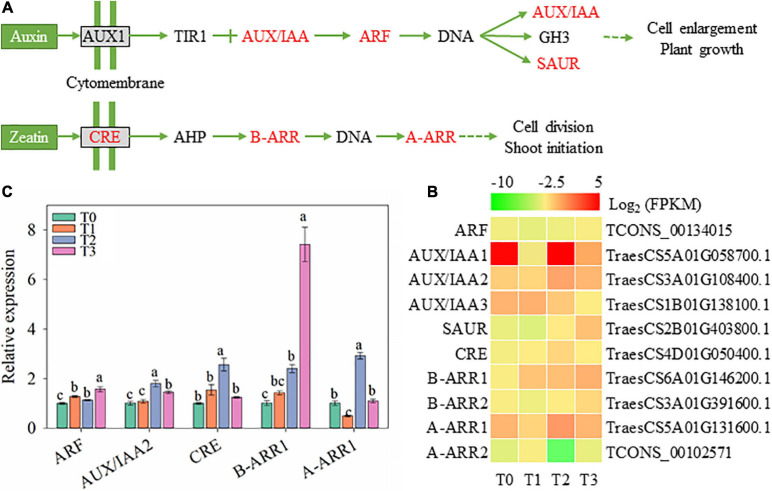
Genes involved in the biosynthesis and signal transduction of IAA and cytokinins. ARF, auxin response factor; SAUR, small auxin-up RNA; CRE, cytokinin receptor; B-ARR, type-B Arabidopsis response regulator; A-ARR, type-A Arabidopsis response regulator. **(A)** Pathway map of the biosynthesis and signal transduction pathways of auxins, cytokinins, and ABA. **(B)** Heatmap of differentially expressed mRNAs in this pathway. **(C)** Verification of the expression of some genes in the pathway. Data are the mean values of four independent replicates (*n* = 4), and error bars show the standard error. Duncan’s multiple-comparison test was used for ANOVA, and different lowercase letters indicate a significant difference at the level of *P* < 0.05). T0, T1, T2, and T3 means the sample was harvested at 0, 2, 24, and 72 h after mowing, respectively.

During the regeneration of wheat seedlings, the signal transduction of IAA and cytokinin might be affected according to changed gene expression in Figure 9. The gene expression of signal molecule related to IAA signal transduction, including AUX/IAA, auxin response factor (ARF), and small auxin-up RNA (SAUR), also increased after mowing (Figure 9B). The expression of *ARF* and *AUX/IAA2* increased within 72 h after mowing according to qRT-PCR (Figure 9C). However, endogenous IAA concentration is decreased during wheat regeneration (shown in Figure 2), which is not completely consistent with the gene expression of signal molecule. The gene expression of signal molecules related to cytokinin, including *CRE* (encoding cytokinin receptor), *B-ARR1*∼*2* (encoding type B *Arabidopsis* response regulator), and *A-ARR1*∼*2* (encoding type A *Arabidopsis* response regulator), changed within 72 h after mowing (Figures 9B,C). The expression of *CRE*, *B-ARR1*, and *A-ARR1* measured by qRT-PCR is consistent with RNA sequencing (Figures 9B, C). These results indicate that auxins and cytokinins may be involved in the post-mowing regeneration of wheat seedlings.

### Interaction Analysis Between DE mRNAs and DE LncRNAs

To determine whether DE lncRNAs interact with DE mRNAs, we performed antisense lncRNA prediction, *cis*-regulation analysis, and co-expression analysis between lncRNAs and mRNAs. The analysis results are shown in Supplementary Table 5; 274 DE lncRNAs interacted with 228 DE mRNAs. No antisense DE lncRNAs were predicted, and 22 DE lncRNAs were identified as *cis*-regulating the expression of 22 DE mRNAs. A total of 149 DE lncRNAs were negatively co-expressed with 67 DE mRNAs, and 193 lncRNAs were positively co-expressed with 145 DE mRNAs. Furthermore, KEGG enrichment of DE target mRNAs is shown in Supplementary Figure 3; a total of 67 DE mRNAs were annotated by KEGG analysis. The proteins they encode are involved in various pathways, including galactose metabolism, starch and sucrose metabolism, plant-pathogen interaction, photosynthesis–antenna proteins, phenylalanine metabolism, the pentose phosphate pathway, fatty acid elongation, protein processing in the endoplasmic reticulum, carbon metabolism, metabolic pathways, carbon fixation in photosynthetic organisms, the MAPK signaling pathway, glyoxylate and dicarboxylate metabolism, glutathione metabolism, endocytosis, RNA transport, spliceosomes, plant hormone signal transduction, phenylpropanoid biosynthesis, and biosynthesis of secondary metabolites.

**FIGURE 10:**
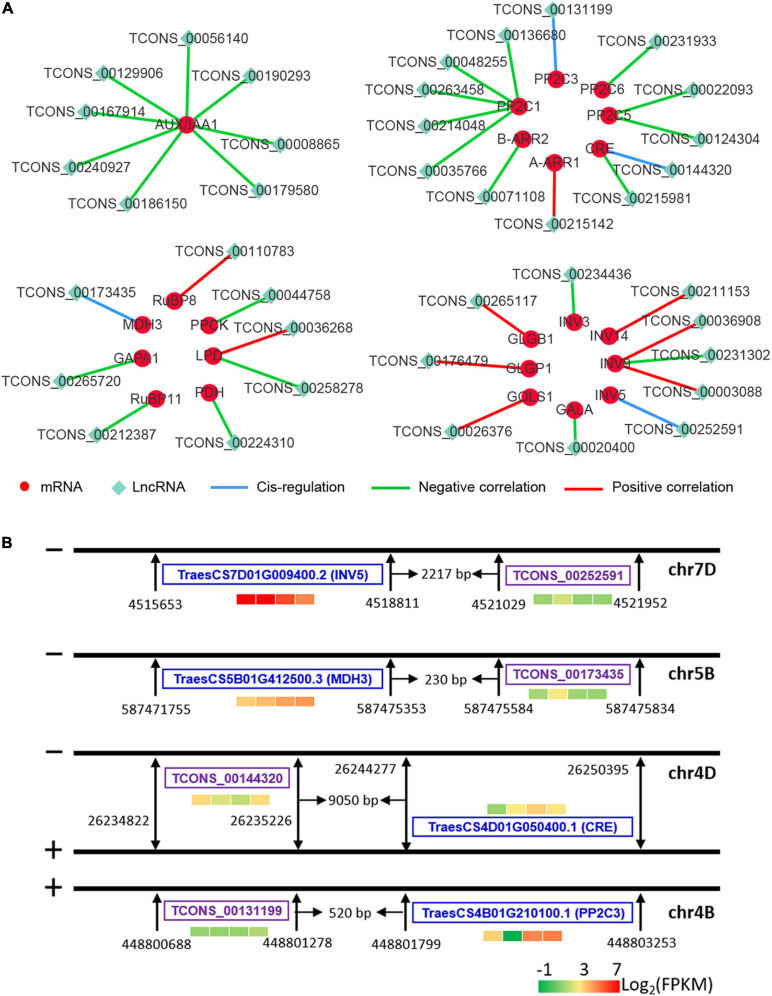
Interaction between mRNAs and lncRNAs. Red and green nodes represent mRNAs and lncRNAs, respectively. Blue edges represent the cis-regulation by lncRNAs of their neighboring mRNAs. Green and red edges represent negative and positive correlations between lncRNAs and mRNAs, respectively. *PP2C*, protein phosphatase 2C; *B-ARR*, type-B *Arabidopsis* response regulator; *A-ARR*, type-A *Arabidopsis* response regulator; *CRE*, cytokinin receptor; *MDH*, malate dehydrogenase; *GAPA*, glyceraldehyde-3-phosphate dehydrogenase; *RuBP*, ribulose bisphosphate carboxylase; *PPCK*, phosphoenolpyruvate carboxykinase; *LPD*, dihydrolipoyl dehydrogenase; *GLGB*, 4-α-glucan branching enzyme; *GLGP*, amylophosphorylase; *GALA*, α-galactosidase; *GOLS*, inositol 3-α-galactosyltransferase; *INV*, β-fructofuranosidase. T0, T1, T2, and T3 means that the sample was harvested at 0, 2, 24, and 72 h after mowing, respectively.

The interactions between some key DE lncRNAs and DE mRNAs are shown in Figure 10. TCONS_00252591, TCONS_00173435, TCONS_00144320, and TCONS_00131199 were predicted to *cis*-regulate the expression of *INV5*, *MDH3*, *CRE*, and *PP2C3* (encoding protein phosphatase 2C), respectively (Figure 10B). The expression levels of *AUX/IAA1*, *PP2C1*, *PP2C5*, *PP2C6*, *CRE*, *B-ARR2*, *GAPA1*, *RuBP11*, *PDH*, *PPCK*, *INV3*, and *GALA* were negatively correlated with their target lncRNAs, whereas the expression levels of *A-ARR1*, *RuBP8*, *GLGB1*, *GLGP1*, *GOLS1*, and *INV14* were positively correlated with their target lncRNAs (Figure 10A and Supplementary Figure 4). Additionally, the expression of dihydrolipoyl dehydrogenase (*LPD*) was found to be positively and negatively related to TCONS_00036268 and TCONS_00258278, respectively (Figure 10A and Supplementary Figure 3). The expression of TCONS_00036908 and TCONS_00003088 was positively correlated with *INV9* expression, whereas the expression of TCONS_00231302 was negatively correlated with *INV9* (Figure 10A and Supplementary Figure 4).

## Discussion

Dual-purpose use of winter wheat for grain and forage production improves land use efficiency and plays an important role in providing winter forage worldwide (Arzadun et al., 2006; Tian et al., 2012; Giunta et al., 2015; Kim and Anderson, 2015). Compensatory regeneration of wheat seedlings after mowing or grazing is a key factor for dual-purpose use, and excessive grazing or mowing of forage wheat seedlings will limit grain yield in the following harvest season (Arzadun et al., 2006). Studying the molecular mechanism of wheat regeneration post-mowing will aid the development of dual-purpose wheat varieties with a high post-mowing regeneration capacity. Therefore, this study identified mRNAs and lncRNAs related to post-mowing regeneration of wheat seedlings. The results showed that some key mRNAs and lncRNAs participated in carbohydrate degradation and plant hormone signal transduction, which might play an important role in post-mowing regeneration of wheat seedlings.

Post-mowing regeneration of wheat is a process of morphogenesis of young leaves, and long-chain coding and non-coding RNAs play an important role in plant development and morphogenesis (Yamada, 2017). In this study, a total of 5882 mRNAs and 672 lncRNAs were identified as differentially expressed in comparison groups (Supplementary Tables 2, 3), suggesting their possible role in the post-mowing regeneration of winter wheat. Furthermore, the number of DE mRNAs and lncRNAs at different regeneration stages differed (Figures 1, 3). In all comparison scenarios, the number of DE mRNAs in the T1/T0 group was the lowest, which was consistent with the lack of visible regeneration of post-mowing wheat seedlings, whereas the number of DE lncRNAs in the T1/T0 group ranked third (Figures 1, 3). Such lncRNAs influence the transcription of downstream genes by interacting with promoters, *cis*-acting elements, or other regulatory activities (Zhang et al., 2017). This result suggested a potential regulatory effect of lncRNAs on the expression of mRNAs in early post-mowing regeneration of wheat seedlings. As mowed wheat seedlings regenerated, the number of DE mRNAs and DE lncRNAs both increased, which was consistent with the visible growth of mowed wheat leaves (Figures 1, 3), indicating that mRNAs and lncRNAs are involved in the late post-mowing regeneration of wheat seedlings.

Carbohydrates are the metabolic source of the materials and energy needed for plant growth; thus, they play an important role in plant morphogenesis (Hill, 1998; Turner et al., 2007). The post-mowing regeneration of wheat seedlings involves the morphogenesis of young leaves and requires large amounts of energy and materials to maintain the division and expansion of leaf cells. Cutting height is one of the most important factors affecting the post-mowing regeneration of wheat seedlings and subsequent grain production (Arzadun et al., 2006). According to previous studies, differences in cutting height result in differences in the total sugar content in the stubble, which ultimately affect post-mowing regeneration and grain production (Arzadun et al., 2006; Turner et al., 2007); similar results were observed in this study. During the post-mowing regeneration of wheat seedlings, the carbohydrate content are changed, as well as the expression of genes related to carbohydrate metabolism (Figures 2, 5–7). BAM and BGL have been reported as key enzymes for the degradation of starch and cellulose (Hill, 1998). The increased expression of their genes suggested that starch and cellulose were degraded to glucose within 72 h after mowing (Figures 2, 5, 6). The expression of 22 genes encoding INV, a key enzyme for sucrose degradation (Ruan, 2014), underwent tremendous changes (Figure 5), suggesting carbohydrate mobilization. SUS, TPP, and TPS are key enzymes involved in trehalose biosynthesis, and trehalose 6-phosphate is also a signal and homeostatic regulator of sucrose levels in plants (Müller et al., 1999; Ruan, 2014; Fichtner and Lunn, 2021). Their significantly increased gene expression suggested the degradation of sucrose and the synthesis of trehalose (Figures 5, 6). Additionally, the expression of genes encoding key enzymes related to the degradation of stachyose, raffinose, galactose, and melibiose, including GALA, GOLS, and RFS (Stick, 2001), was significantly increased during the post-mowing regeneration of wheat seedlings (Figure 6). Changes in the gene expression and carbohydrate concentration in these pathways suggested that carbohydrates in the wheat stubble had been mobilized. The carbohydrates in the stubble were degraded into glucose and fructose, which were used for the regeneration of young leaves by the mowed wheat seedlings. This was consistent with a physiological experiment (Figure 2) and a previous report (Arzadun et al., 2006; Turner et al., 2007). One most important physiological function of glycolysis is to provide energy and carbon skeletons for plant growth and development (Sung et al., 1988; Plaxton, 1996). HXK, PFK, ALDO, PDC, PGK, PPDK, PEPC, and MAE are important enzymes in glycolysis and the Krebs cycle (Plaxton, 1996), and their gene expression in stubble was increased during post-mowing regeneration. The increased expression of these genes may result in enhanced glycolytic activity, ultimately providing more ATP and carbon skeletons for the Krebs cycle and the biosynthesis of amino acids during the post-mowing regeneration of wheat seedlings (Figure 7). All these changes results in carbohydrate mobilization and may be helpful for the post-mowing regeneration of wheat. The result is consistent with a previous study (Turner et al., 2007).

The Calvin cycle is an important component of photosynthesis, and enhanced Calvin cycle activity can fix more CO_2_ to produce more carbohydrates during post-mowing regeneration of wheat seedlings. In this study, significantly increased expression of genes involved in the Calvin cycle might enhance the fixation of CO_2_ (Figure 8); such genes included *RuBP1–18*, *GAPA1–4*, and *ALDO1–4*, which encode key enzymes for CO_2_ fixation (Lu et al., 2012). The increased gene expression of these enzymes in this study suggested possible increased fixation of CO_2_ and the provision of more carbon for the regeneration of mowed wheat seedlings (Figure 8). This possible increased carbon storage appeared to be the material basis for carbohydrate synthesis and the regeneration of mowed young leaves in winter wheat. It may be another favorable factor for the post-mowing regeneration of seedlings.

Plant hormones are key factors regulating plant morphogenesis (Du et al., 2018; Ali et al., 2020) and may play an important role during post-mowing regeneration of wheat seedlings. The interaction of endogenous auxins and cytokinins affects leaf morphogenesis and involves the complex interplay of a multitude of regulatory pathways (Gonzalez et al., 2012; Bar and Ori, 2014; Skalák et al., 2019). Before mowing, high concentrations of auxins accumulate in the base of the wheat cauloid, which inhibits the activity of KNOX1 and limits CK biosynthesis (Su et al., 2011; Guenot et al., 2012). This is consistent with our observations of auxin and CK content (Figure 2). Wu et al. (2017) reported that shade significantly increased auxin content and resulted in a smaller leaf. This partially confirms that high concentrations of auxin inhibit growth, thus inhibiting the post-mowing regeneration of wheat. After mowing, the CK content increased significantly, and the ratio of CK to auxin also increased (Figure 2). Increased concentrations of CK promote the growth and maintenance of the SAM (Gordon et al., 2009), aiding the regeneration of mowed wheat seedlings; this is similar to the induction of explant buds by cytokinins. Regeneration is a violent process of cell division, and CDKA and CYCD are central to the phase transition of the cell cycle (Gaamouche et al., 2010). Increased CK content can increase the level of CYCD, thereby activating CDKA and accelerating the cell cycle (Inzé and De Veylder, 2006; Perrot-Rechenmann, 2010). In addition, CK controls the transition from cell division to cell expansion and stimulates cell expansion and differentiation during the cell expansion phase (Skalák et al., 2019). This is consistent with our observation of an increased number of regenerated wheat leaves (Supplementary Video 1). The increased CK concentration and high CK/auxin ratio may lead to the fast regeneration of mowed wheat leaves. Therefore, auxins, cytokinins, and their interplay potentially play an important role in the morphogenesis of leaves in mowed wheat seedlings. In addition, the crosstalk between hormone and sugar signaling may participate in post-mowing regeneration of wheat. Mounting evidence suggests interplay between hexose and auxin signaling in mediating cell proliferation, cell expansion, and seed development (Wang and Ruan, 2013). The degradation of starch produces abundant hexoses, such as glucose, which inhibits auxin signaling and facilitate cell expansion (Mishra et al., 2009). In this study, sugar and auxin content were changed, which is similar to previous studies and may be a factor of the regeneration of mowed wheat. In addition, high concentrations of CK can stimulate sugar and energy metabolism and increase the expression of *FPB*, *GPI*, *MDH*, and *BGL*, result in increased soluble sugar, increase turgor pressure and cell wall expansion as shown in previous studies (Cosgrove, 2000; Skalák et al., 2019). This may be another factor to promote the post-mowing regeneration of wheat seedlings.

In plants, lncRNAs have been reported to regulate gene expression at multiple levels by a number of complex mechanisms, including transcriptional regulation, post-transcriptional regulation, and epigenetic modifications (Chekanova, 2015; Liu et al., 2015). This regulation of gene expression can occur either *cis* or *trans* to the lncRNA by sequence complementarity or homology with RNAs or DNA (Kung et al., 2013; Yang et al., 2014). In this study, four lncRNAs (TCONS_00173435, TCONS_00131199, TCONS_00144320, and TCONS_00252591) were predicted to *cis*-regulate the expression of *MDH3*, *PP2C3*, *CRE*, and *INV5*, respectively (Figure 10B). However, there was less correlation between the expression trends for these lncRNAs and mRNAs. It was difficult to judge whether lncRNAs interacted with these mRNAs and other regulatory factors that might influence the expression of mRNAs. Additionally, lncRNAs can regulate gene expression of mRNA by collinearity in a variety of mechanisms (Chekanova, 2015; Rai et al., 2018). In this study, most DE lncRNAs interacted with mRNAs in this manner. Nine lncRNAs were positively co-expressed with their target mRNAs (Figure 10A) and might regulate the expression of target mRNAs by enhancing expression of the transcription complex (Zhang et al., 2017). Most of the lncRNAs were negatively co-expressed with their target mRNAs (Figure 10A) and might regulate their target mRNAs by acting as miRNA target mimics as shown by previous study (Rai et al., 2018). In all cases, DE target genes included *AUX/IAA1*, *PP2C*s, *B-ARR2*, *A-ARR1*, *CRE*, *MDH3*, *GAPA1*, *RuBP*s, *PDH*, *LPD*, *PPCK*, *GLGP1*, *GLGB1*, *GLGS1*, *GALA*, and *INV*s. Furthermore, expression levels of *INV3*, *INV5*, *INV9*, *INV14*, *GOLS1*, *GLGP1*, and *AUX/IAA1* were higher than those of their differentially expressed homologous genes, indicating that these lncRNAs might affect their expression (Supplementary Figure 4). These results indicate that lncRNAs may be involved in the regulation of regeneration in mowed wheat seedlings. Although we discovered that sugars and phytohormones were involved in post-mowing regeneration of wheat, there are some limitations to the conclusions that can be drawn from these results. Further experiments are needed to determine how phytohormones regulate post-mowing regeneration of seedlings and to verify the interaction between lncRNAs and targeted genes.

In addition, the molecular response of post-mowing regeneration must be distinguished from stress responses induced by mechanical damage. In the experiment, parts above 3 cm of wheat seedlings were mowed, but only 2 cm above the ground was sampled. The cut wound was not sampled and this reduces the influence of the injury response on the experimental results to a certain extent. But the mowing during sampling and wound signal conduction will still affect the experimental results. Even it may be that the mowing induced the plant’s regeneration signal. Because mere mechanical damage can also induce changes in carbohydrate and hormone content (Chandra et al., 2012; Lukaszuk et al., 2017). In Furthermore, mechanical damage changes the activities of sucrose hydrolysis-related enzymes (Lukaszuk et al., 2017). However, mechanical damage induced hormonal response such as ethylene, jasmonic acid, and abscisic acid (Cheong et al., 2002; Savatin et al., 2014), while regeneration was induced by cytokinin and auxin in this study. Therefore, it can be seen that there is a difference between regeneration and injury response after mowing.

Post-mowing regeneration of seedlings is a key factor in the suitability of winter wheat for dual-purpose use. This study examined the molecular responses of long-chain coding and non-coding RNAs to illuminate the mechanism of regeneration. Function enrichment analysis demonstrated that DE mRNAs were involved in carbohydrate mobilization, photosynthesis, and phytohormone signal transduction. Physiological experiments combined with gene expression verification indicated that carbohydrate mobilization and enhanced photosynthesis provide energy and carbon skeletons for cell proliferation and expansion during post-mowing regeneration of wheat. Combining the changes in auxin and cytokinin content, and gene expression, we draw the following conjecture. The decreased auxin content may relieve the inhibition of cytokinin synthesis, enhance cytokinin biosynthesis, control the transition from cell division to cell expansion, and may stimulate cell expansion and differentiation during the cell expansion phase, and eventually accelerate post-mowing regeneration of seedlings. Furthermore, 39 DE lncRNAs might affect the expression of key genes involved in carbohydrate metabolism and phytohormone signal transduction. The main finding of this study is that carbohydrates and phytohormones are involved in post-mowing regeneration of wheat seedlings, and lncRNAs may play a role in this process. This can serve to guide the breeding of wheat varieties with higher stubble sugar content and the development of external reagents to promote forage regeneration. Our next study will focus on (i) the interaction between phytohormones and carbohydrate metabolism, and (ii) the regulatory effects of lncRNA on the expression of related genes.

## Data Availability Statement

The datasets presented in this study can be found in online repositories. The names of the repository/repositories and accession number(s) can be found below: https://www.ncbi.nlm.nih.gov/, PRJNA638505 (SRR11973944, SRR11973945, SRR11973946, SRR11973947, SRR11973948, SRR11973949, SRR11973950, SRR11973951, SRR11973952, SRR11973953, SRR11973954, and SRR11973955).

## Author Contributions

YX and CZ designed and directed this study and drafted the manuscript. GC performed the experiments, analyzed the data, and revised the manuscript. MZ determined the expression of mRNAs by qRT-PCR. HT investigated the regeneration of wheat and completed carbohydrate determination. ZW and MM determined the levels of endogenous auxins and cytokinins. SL improved the data analysis and revised the manuscript. All authors contributed to the article and approved the submitted version.

## Conflict of Interest

HT was employed by Shaanxi Province Seed Industry Group Co., Ltd. The remaining authors declare that the research was conducted in the absence of any commercial or financial relationships that could be construed as a potential conflict of interest.

## Publisher’s Note

All claims expressed in this article are solely those of the authors and do not necessarily represent those of their affiliated organizations, or those of the publisher, the editors and the reviewers. Any product that may be evaluated in this article, or claim that may be made by its manufacturer, is not guaranteed or endorsed by the publisher.

## Footnotes

1
ftp://ftp.ncbi.nlm.nih.gov/genbank/

2
https://wheat-urgi.versailles.inra.fr/

3
http://cpc.cbi.pku.edu.cn/

4
http://www.r-project.org/

5
http://www.omicshare.com/tools

6
http://www.tbi.univie.ac.at/RNA/RNAplex.1.html

7
https://cytoscape.org/

## Supplementary Material

Supplementary Figure 1 Standard curve of indole-3-acetic acid (IAA), indole-3-carboxaldehyde, and cis-zeatin (cZ).

Supplementary Figure 2 Trend clustering analysis of differentially expressed mRNAs and KEGG enrichment analysis of mRNAs in different classes. **(A)** Trend clustering analysis of differentially expressed mRNAs. **(B–D)** KEGG enrichment of mRNAs in classes I, II, and III. The expression of mRNAs in class I was changed at 2 h but not at 24 and 72 h after mowing. The expression of mRNAs in class II did not change at 2 h but changed at 24 or 72 h after mowing. The expression of mRNAs in this class was altered at 2, 24, and 72 h after mowing.

Supplementary Figure 3 KEGG enrichment of differentially expressed targeted mRNAs of differentially expressed lncRNAs.

Supplementary Figure 4 Correlation analysis of the expression of lncRNAs and their target mRNAs. The horizontal and vertical coordinates represent the FPKM values of mRNAs and lncRNAs, respectively. T0, T1, T2, and T3 means the sample was harvested at 0, 2, 24, and 72 h after mowing, respectively.

Supplementary Table 1 Primers for qRT-PCR.

Supplementary Table 2 All identified differentially expressed lncRNAs and annotation in GO and KEGG analysis.

Supplementary Table 3 All identified differentially expressed mRNAs and their annotation in GO and KEGG database.

Supplementary Table 4 Expression trend cluster analysis of differentially expressed mRNAs.

Supplementary Table 5 Interaction analysis between differentially expressed mRNAs and differentially expressed lncRNAs.

Supplementary Video 1 Indoor simulation of post-mowing regeneration of wheat. The wheat seedlings were placed in a growth chamber at a temperature of 15/20°C and a 10/14 h dark/light cycle.
