# Supplementary figures and images for: RNA Sequencing Reveals Dynamic Carbohydrate Metabolism and Phytohormone Signaling Accompanying Post-mowing Regeneration of Forage Winter Wheat (Triticum aestivum L.)
Source: Front Plant Sci. 2021 Jul 29;12:664933. doi: 10.3389/fpls.2021.664933 (PMC8358837; doi:10.3389/fpls.2021.664933)

cZ

Peak Area

$$\hat{y} = 194677.4461x - 3094.859, R^2 = 1$$

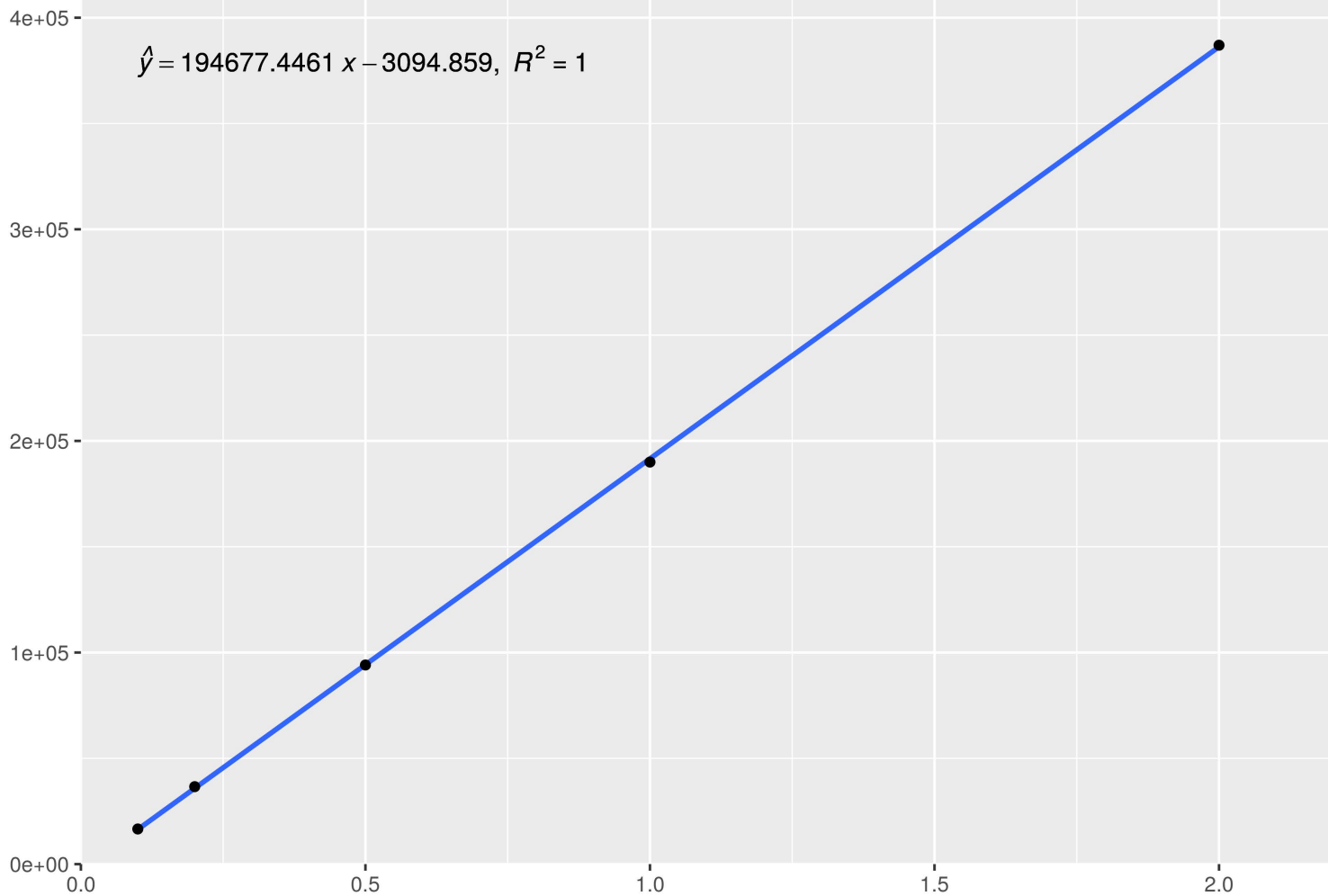

Standard Concentration (ng/mL)

# IAA

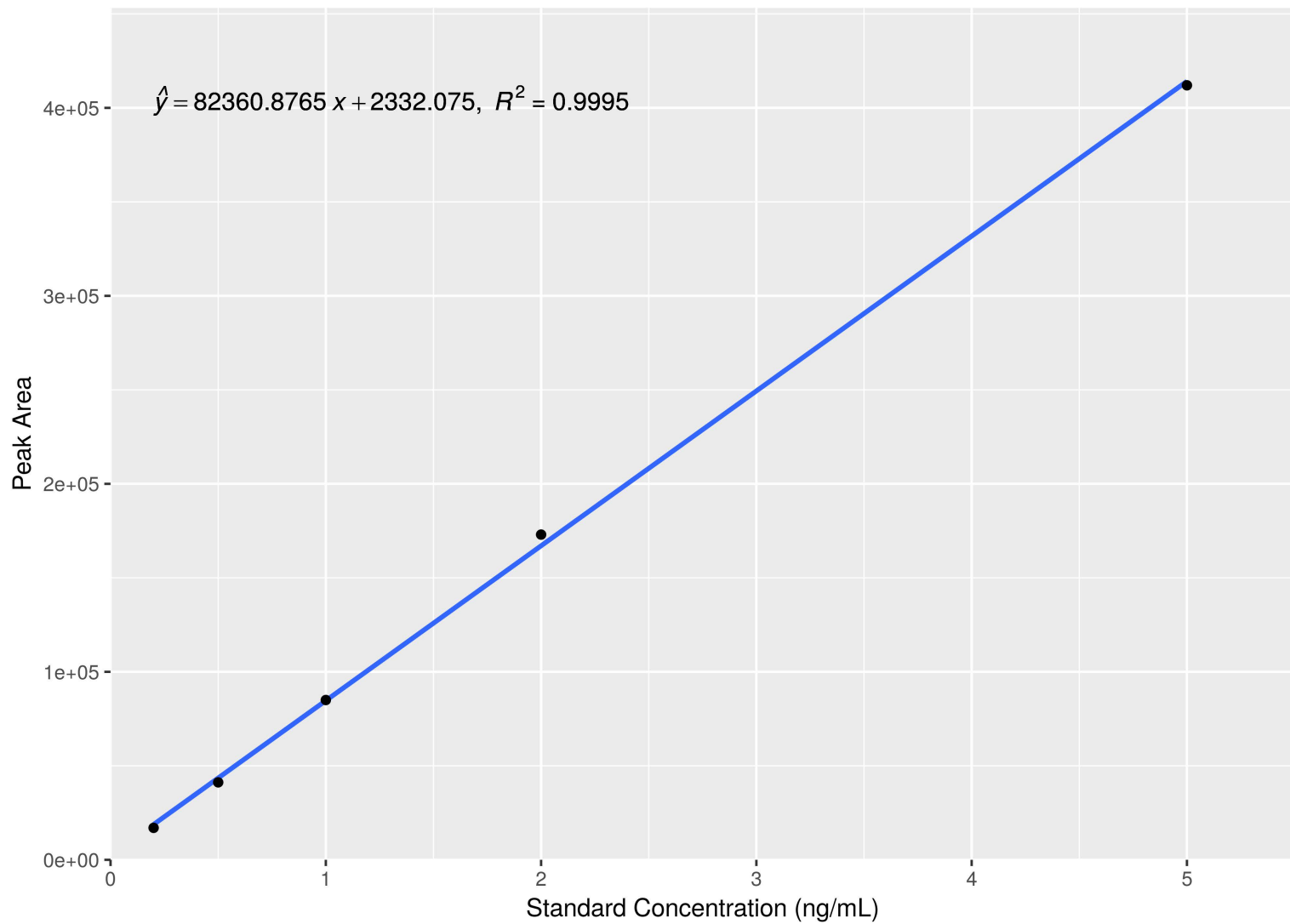

## ICA

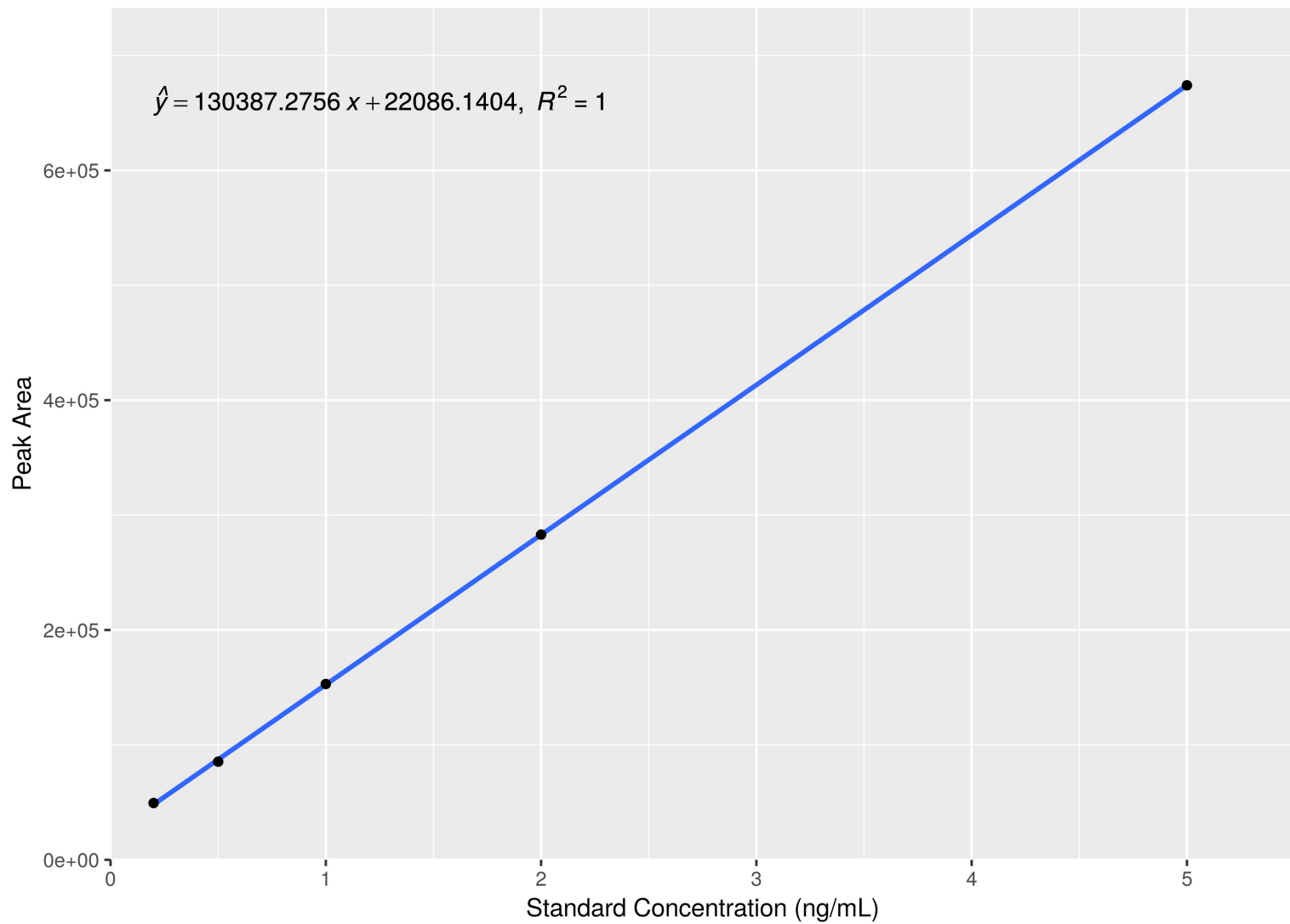

Supplement: Supplementary Figure 1 — Standard curve of indole-3-acetic acid (IAA), indole-3-carboxaldehyde, and cis-zeatin (cZ). [file Data_Sheet_1.PDF]

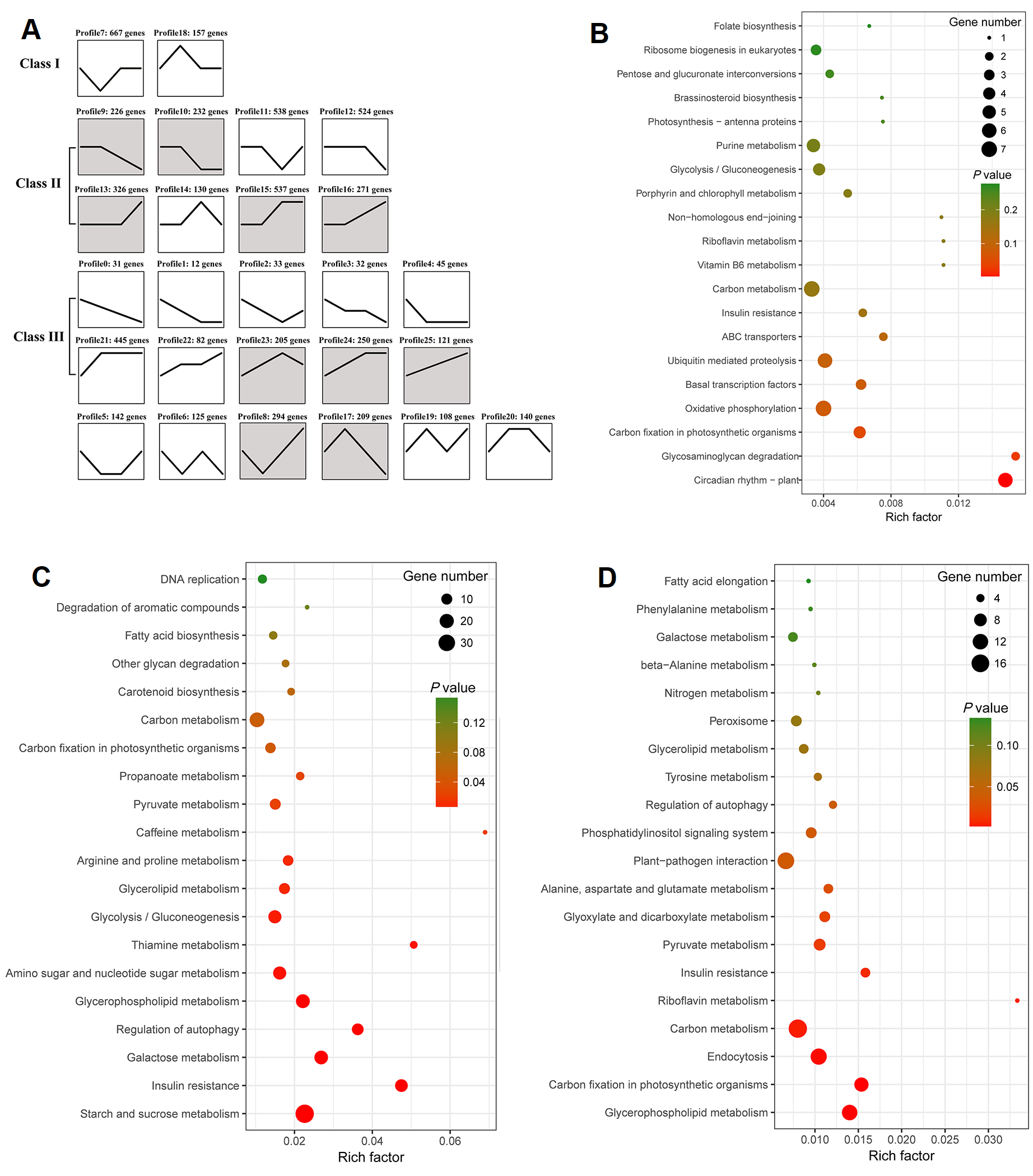

Supplement: Supplementary Figure 2 — Trend clustering analysis of differentially expressed mRNAs and KEGG enrichment analysis of mRNAs in different classes. (A) Trend clustering analysis of differentially expressed mRNAs. (B–D) KEGG enrichment of mRNAs in classes I, II, and III. The expression of mRNAs in class I was changed at 2 h but not at 24 and 72 h after mowing. The expression of mRNAs in class II did not change at 2 h but changed at 24 or 72 h after mowing. The expression of mRNAs in this class was altered at 2, 24, and 72 h after mowing. [file Image_1.TIF]

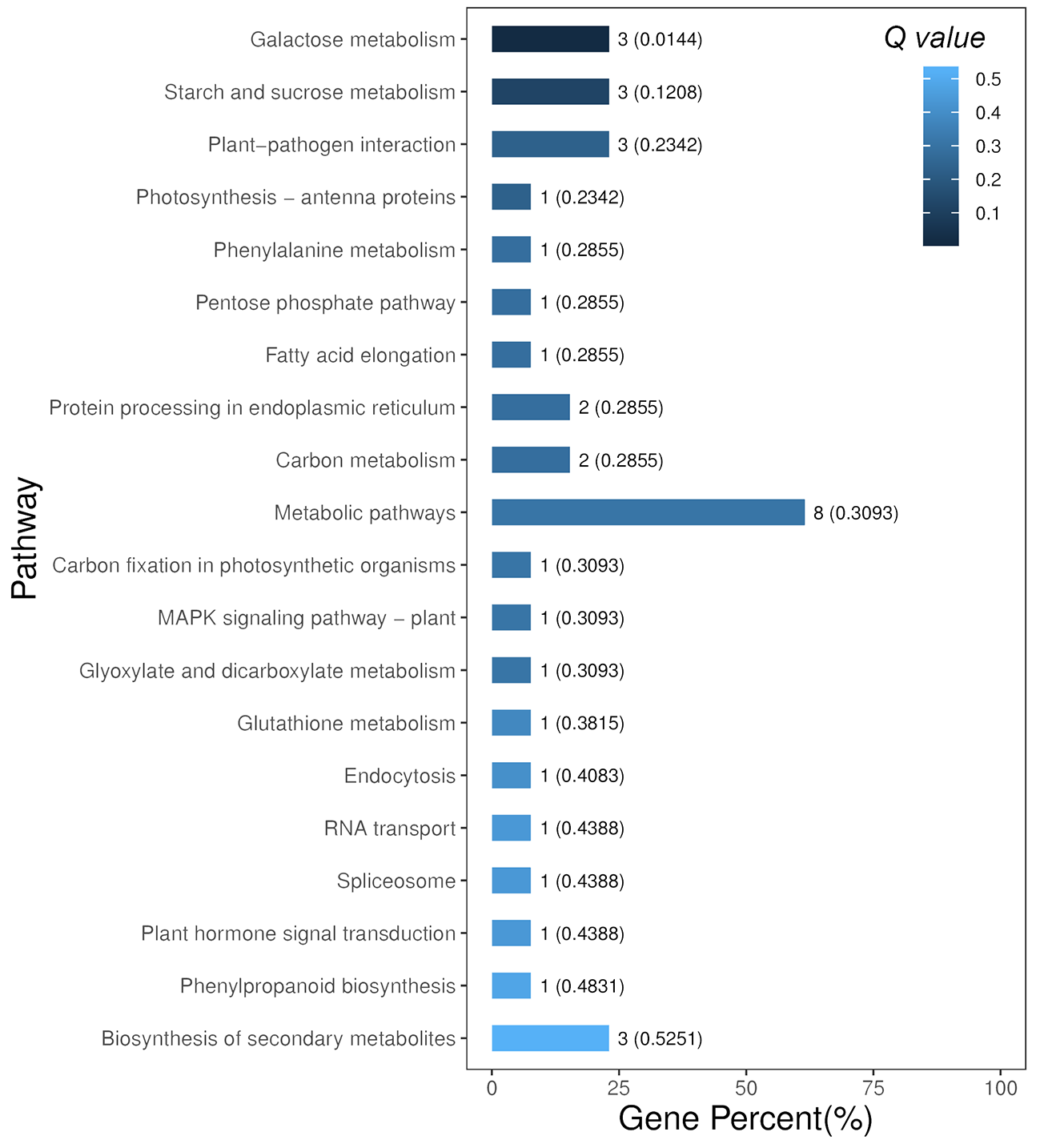

Supplement: Supplementary Figure 3 — KEGG enrichment of differentially expressed targeted mRNAs of differentially expressed lncRNAs. [file Image_2.TIF]

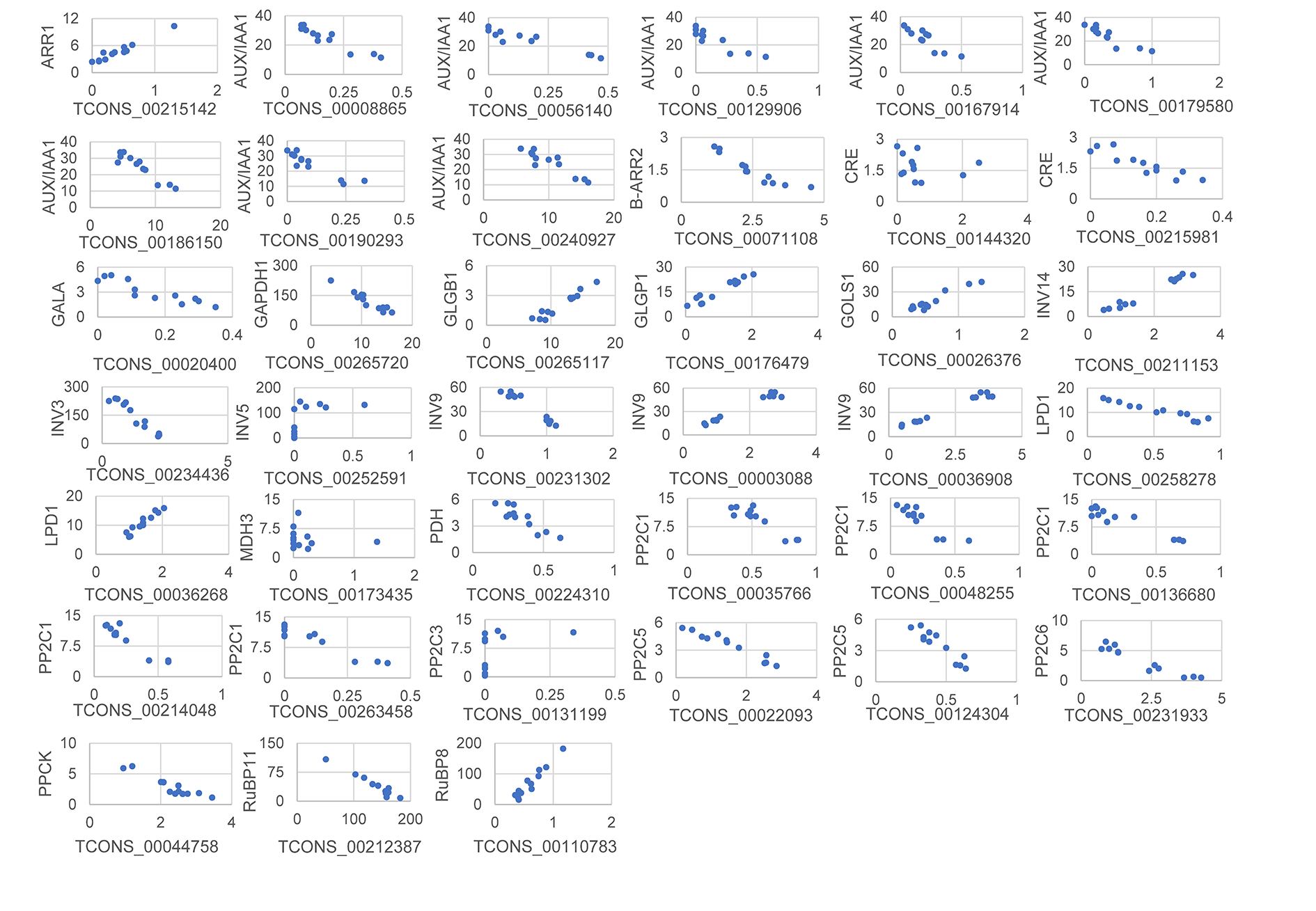

Supplement: Supplementary Figure 4 — Correlation analysis of the expression of lncRNAs and their target mRNAs. The horizontal and vertical coordinates represent the FPKM values of mRNAs and lncRNAs, respectively. T0, T1, T2, and T3 means the sample was harvested at 0, 2, 24, and 72 h after mowing, respectively. [file Image_3.TIF]
